# Shaping the future of gastrointestinal cancers through metabolic interactions with host gut microbiota

**DOI:** 10.1016/j.heliyon.2024.e35336

**Published:** 2024-07-27

**Authors:** Wen Xie, Aditi Sharma, Hitesh Kaushik, Lalit Sharma, Nistha, Md Khalid Anwer, Monika Sachdeva, Gehan M. Elossaily, Yingbo Zhang, Ramkumar Pillappan, Maninderjit Kaur, Tapan Behl, Bairong Shen, Rajeev K. Singla

**Affiliations:** a Department of Pharmacy and Institutes for Systems Genetics, Center for High Altitude Medicine, Frontiers Science Center for Disease-related Molecular Network, West China Hospital, Sichuan University, Chengdu, Sichuan, 610041, China; b School of Pharmaceutical Sciences, Shoolini University, Solan, H.P, 173229, India; c Department of Pharmaceutics, College of Pharmacy, Prince Sattam Bin Abdulaziz University, Alkharj, Saudi Arabia; d Fatima College of Health Sciences, Al Ain, United Arab Emirates; e Department of Basic Medical Sciences, College of Medicine, AlMaarefa University, P.O. Box 71666, Riyadh, 11597, Saudi Arabia; f Nitte (Deemed to be University), NGSM Institute of Pharmaceutical Sciences, Mangaluru, Karnataka, India; g Department of Pharmaceutical Sciences, lovely Professional University, Phagwara, India; h Amity School of Pharmaceutical Sciences, Amity University, Sahibzada Ajit Singh Nagar, Punjab, India; i School of Pharmaceutical Sciences, Lovely Professional University, Phagwara, Punjab, 1444411, India; j Institutes for Systems Genetics, West China Tianfu Hospital, Frontiers Science Center for Disease-related Molecular Network, West China Hospital, Sichuan University, Chengdu, Sichuan, 610218, China

**Keywords:** Gastrointestinal cancers, Microbiome research, Metabolic interplay, Precision diagnosis, Therapeutic approaches, Microbiome

## Abstract

Gastrointestinal (GI) cancers represent a significant global health challenge, driving relentless efforts to identify innovative diagnostic and therapeutic approaches. Recent strides in microbiome research have unveiled a previously underestimated dimension of cancer progression that revolves around the intricate metabolic interplay between GI cancers and the host's gut microbiota. This review aims to provide a comprehensive overview of these emerging metabolic interactions and their potential to catalyze a paradigm shift in precision diagnosis and therapeutic breakthroughs in GI cancers. The article underscores the groundbreaking impact of microbiome research on oncology by delving into the symbiotic connection between host metabolism and the gut microbiota. It offers valuable insights into tailoring treatment strategies to individual patients, thus moving beyond the traditional one-size-fits-all approach. This review also sheds light on novel diagnostic methodologies that could transform the early detection of GI cancers, potentially leading to more favorable patient outcomes. In conclusion, exploring the metabolic interactions between host gut microbiota and GI cancers showcases a promising frontier in the ongoing battle against these formidable diseases. By comprehending and harnessing the microbiome's influence, the future of precision diagnosis and therapeutic innovation for GI cancers appears more optimistic, opening doors to tailored treatments and enhanced diagnostic precision.

## Graphical abstract

**Figure:**
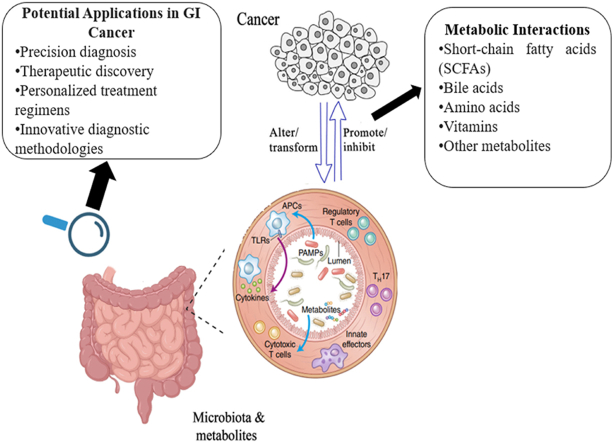

## 1 Introduction

Gastrointestinal (GI) cancer begins in the GI tract, which includes the organs and structures involved in food digestion [1]. These malignancies can affect the GI tract in various parts, including the small intestine, large intestine (colon and rectum), stomach, liver, pancreas, esophagus, and anus. It has been reported that the human oral microbiota plays a vital role in developing GI cancer. Oral microbiota includes these major phyla: Firmicutes, Proteobacteria, Bacteriodetes, Fusobacteria, and Actinobacteria. In the oral cavity, enrichment of archaea, including *Methanobrevibacter oralis, Methnanobacterium curvum/congolese*, and *Methanosarcina mazeii,* are associated with oral cancers [2]. GI malignancies account for a sizable fraction of cancer incidence and deaths worldwide, according to the IARC-2020 [3]. The rates of occurrence and fatality of GI cancers vary across regions and populations, with higher prevalence in specific geographical areas. For example, Eastern Asia has among the highest rates of stomach cancer occurrence, while colorectal cancer is more common in Western countries [4].

Gastric cancer is one of many GI malignancies, which is a product of several risk factors, including behavioral risks like smoking and alcohol intake, body mass index (BMI), and some infectious agents such as Hepatitis B and C viruses [5]. The gut microbiome plays a vital role in both health and illness. It refers to the diverse community of microorganisms that live within the GI system, including viruses, bacteria, fungi, and other microbes [6]. Regarding digestion and nutrient absorption, the gut microbiome significantly contributes to our ability to process complex carbohydrates and dietary fibers [7]. The breakdown produces short-chain fatty acids (SCFAs), critical ATP sources [8].

Additionally, the microbiota produces enzymes that assist in the digestion of otherwise indigestible substances [9]. Furthermore, the gut microbiome extends its influence on immune system modulation. It actively investigates and modifies the host's immune system, influencing intake and adaptive immunological responses [10]. Conversely, dysbiosis, signifying a non-equilibrium in the gut microbiota, can compromise immunological tolerance, potentially precipitating autoimmune conditions [11]. GI health is also closely related to a balanced gut microbiota. It plays a pivotal role in protecting against GIT disorders, such as inflammatory bowel disease (IBD), by upholding gut barrier integrity [12]. Moreover, commensal bacteria within the GI contribute to the GI's well-being by producing antimicrobial peptides and mucus [13].

Metabolic health is another area significantly impacted by the gut microbiome. It plays a role in shaping metabolic processes, encompassing the regulation of energy storage and the activation of adipose tissue [14]. Additionally, dysbiosis is linked to metabolic conditions such as obesity and type II diabetes [15,16]. Intriguingly, emerging research on the gut microbiota indicates its association with mood, behavior, and even neurological conditions [17,18]. It also shows the broad influence of gut microbiota on emotional health and thinking capacity. In addition, colonizing the gut microbiota influences allergy and even autoimmunity via effects on pro-inflammation vs anti-inflammation [19,20].

Furthermore, in the case of infectious disorders, commensal bacteria play a protective role by occupying niches and producing antimicrobial compounds against pathogens [21]. Conversely, antibiotic-induced dysbiosis can increase susceptibility to infections like *Clostridium difficile* [22]. Various interventions are being explored, considering the therapeutic potential of gut microbiome modification. These include probiotics, prebiotics, and fecal microbiota transplantation (FMT) to improve health and cure various illnesses [[23], [24], [25]].

Gut microbiota, a diverse community of bacteria in the digestive unit, has been recognized as a critical component for many facets of health and disease. It is increasingly apparent that the gut microbiota metabolism may be one of the most potent factors for GI cancers genesis, progression and treatment responsiveness. This review explores the intricate metabolic interactions between gut microbiota and GI cancers. By examining these interactions in-depth, this review aims to shed light on their significant implications for precision diagnosis and innovative therapeutic approaches in the context of GI cancers.

## 2 Gut microbiota

### 2.1 Overview of gut microorganisms

**Fig. 1:**
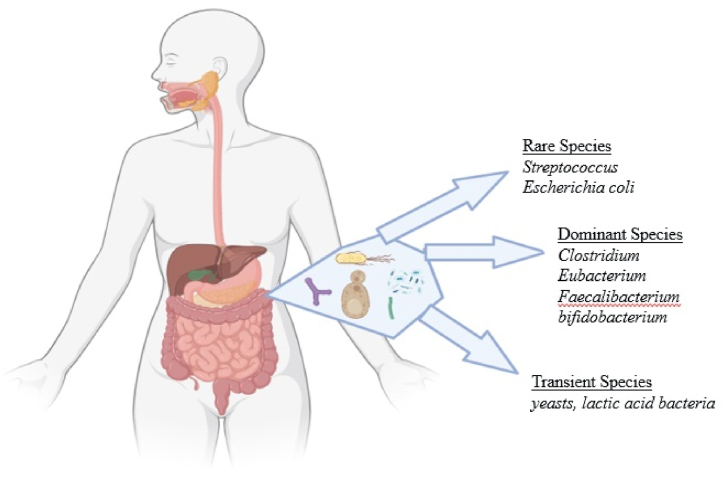
Gut microbiota with different species of bacteria labeled in different regions. Diverse gut bacteria serve distinct digestion functions: food breakdown, vitamin production, and infection defense.

The human GI tract is one of the most extensive interfaces (with a surface area ranging from 250 to 400 square meters) connecting the host, environmental elements, and antigens within the human body [26]. About 60 tons of food pass through the human GI tract throughout an ordinary lifespan, transporting many microorganisms from the environment and presenting a substantial challenge to the gut's integrity [27]. The human gut, a complex and vital part of our digestive system, is home to trillions of microbes, collectively called gut microbiota [6]. These microorganisms include bacteria, viruses, fungi, and others, forming a dynamic and diverse ecosystem within our intestines (Fig. 1) [28]. In the past few years, investigations into the gut microbiota have revealed its pivotal function in upholding general health and wellness.

The human GI microbiome is a remarkably diverse and intricate ecosystem comprising various microorganisms, including viruses, archaea, bacteria, and fungi [7]. This intricate composition and structure have been extensively investigated, with valuable insights drawn from credible sources [29].

### 2.2 Functional roles of the gut microbiota

The human GI tract is a thriving habitat for many microorganisms, primarily bacteria [30]. With an estimated presence of over 1000 distinct bacterial species, the gut emerges as a remarkable ecosystem in its own right [31]. Among these bacterial inhabitants, Bacteroidetes and Firmicutes are the most common phylum, followed by Proteobacteria and Actinobacteria, further contributing to the GI microbiota's complexity [32]. Nevertheless, the intrigue extends beyond bacteria alone, encompassing the realm of viruses and bacteriophages, which coexist as integral constituents of the gut microbiota, wielding the ability to influence bacterial populations [33]. Moreover, the microbiota's diversity extends to the realms of fungi and archaea and, through these, has garnered comparatively less extensive scrutiny in the context of the GI ecosystem [34]. Noteworthy variations in microbial diversity emerge with the length of the GI tract, with the colon manifesting as a protector of higher diversity when juxtaposed with the small intestine [35]. However, this diversity is far from static; it is subject to fluctuations among individuals and across time frames, under the sway of multifarious factors, including dietary choices, aging, and health status [36]. The function of this dynamic microbial assembly within our GI tract is profound, stretching from digestion and nutrient metabolism to synthesizing vital compounds, including vitamins and other bioactive molecules [37]. Also, the complex microbiota plays an active function in the host's immune system, not only as a passive observer but as a crucial participant, actively influencing and regulating immunological responses [10].

### 2.3 Implications of gut microbiota dysbiosis

**Fig. 2:**
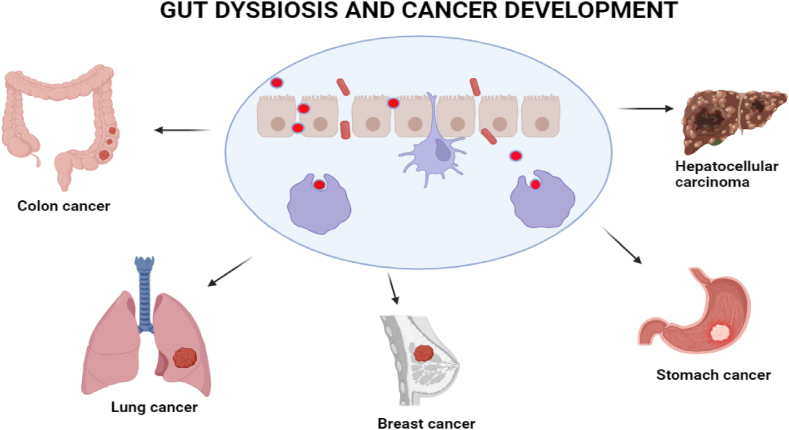
Dysbiosis can cause a variety of malignancies, including breast, colon, lung, stomach, and intestinal tumors.

Dysbiosis, defined as GI microbiota abnormalities, has been linked to a variety of health problems ranging from IBD to obesity and other metabolic disorders [16]. Gut microbiota dysbiosis is also linked with various cancers, as illustrated in Fig. 2. This realization has fuelled a rapidly expanding field of study into the clinical potential of gut microbiota modulation. Dietary treatments, prebiotics, probiotics, and even FMT are all current research fields that promise to address various medical issues [38]. The gut microbiota is vital in sustaining host health in various ways. It actively participates in complex carbohydrate digestion, producing SCFAs such as butyrate, acetate, and propionate. These SCFAs provide a crucial energy source for the host and play a pivotal role in GI health maintenance [7].

Furthermore, gut microbiota is central in regulating the host's immune system, acting as an educator and regulator [39]. This role is crucial in developing invulnerable tolerance and orchestrating rejoinders to invading pathogens, ensuring the host's defense mechanisms remain balanced and effective [10]. In defense, the gut microbiota shields against potential pathogens by fostering a competitive environment [22]. Beneficial gut bacteria are active for resources and attachment sites within the gut, impeding the colonization of harmful microorganisms and serving as a formidable line of defense against infections [40]. Metabolically, gut microbiota exerts a reflective influence on host metabolic processes, contributing to the fine-tuned regulation of energy balance. Dysbiosis has been linked to metabolic illnesses such as insulin resistance and obesity, highlighting the microbiota's significance in health metabolism [15].

Moreover, the gut microbiome operates as a biochemical factory, synthesizing bioactive compounds such as SCFAs. These chemicals have strong anti-inflammatory characteristics and play a pivotal role in maintaining the integrity of the gut lining while regulating immunological responses and immune system function and guaranteeing gut health [41]. Additionally, the gut microbiota contributes to the host's well-being by safeguarding the integrity of the gut barrier. It functions as a team responsible for preserving the barrier, thwarting the undesired movement of harmful substances from the gut's interior into the bloodstream, a mechanism that could incite systemic inflammation and other health complications [42].

Regarding nutrition, the gut microbiota assumes an added responsibility as a producer of vitamins. Specific gut microorganisms are significant in manufacturing vital vitamins such as vitamin K and various B vitamins. This microbial contribution elevates the host's nutritional status, emphasizing the microbiota's contribution to maintaining general well-being [43]. The gut microbiota plays an active role in the complex dialogue between the gut and the brain. This gut-brain axis has been studied, which indicates that the microbiome can impact brain functions and behavior. Interestingly, this relationship between these two promises huge impacts on mental health and welfare in general [17].

## 3 Microbial metabolism

### 3.1 Diversity of microbial metabolism

Microbial metabolism is the intricate web of biochemical processes undertaken by microorganisms, encompassing bacteria, archaea, fungi, and other minuscule life forms, as they seek to acquire the energy and nutrients necessary for their growth, replication, and survival [7]. This multifaceted domain encompasses an elaborate network of chemical reactions and pathways microorganisms employ to extract energy from various substances while concurrently synthesizing essential cellular components vital for survival [44]. Within this realm, microbial metabolism spans a vast array of metabolic pathways, embracing glycolysis, the citric acid cycle, oxidative phosphorylation, fermentation, and a diverse spectrum of biosynthetic routes, encompassing amino acids, nucleotides, and lipids [45]. Microbial metabolism is fundamentally driven by energy generation, often adenosine triphosphate (ATP), facilitated through aerobic and anaerobic respiration alongside fermentation [46].

Nevertheless, the extraordinary diversity of microbial metabolism extends further as different microorganisms harness an assortment of energy sources, including organic compounds, inorganic minerals, and even light through photosynthesis [47]. Moreover, microbial metabolism holds profound implications for environmental biogeochemistry, exerting significant influence over critical biogeochemical cycles, including carbon, nitrogen, and sulfur, thereby playing an essential role in the intricate dance of nutrient cycling and the ecosystem equilibrium [48].

### 3.2 Microbial metabolism in the gut microbiota

Gut microbiota, comprising a vast population of trillions of microorganisms inhabiting the GI tract, profoundly influences host metabolism [30]. Within this intricate microbial ecosystem, gut microbes ferment complex carbohydrates, which, while indigestible by humans, yield SCFAs as valuable byproducts [7]. These SCFAs function as a vital energy supply for the host and wield control over various metabolic processes. Furthermore, gut microbiota contributes significantly to metabolizing bile acids, a critical player in fat absorption and cholesterol homeostasis [15]. In the context of host weight and obesity, dysbiosis has emerged as a pertinent factor; an imbalance in microbial composition has been associated with obesity, with specific microbial species displaying the capacity to improve dietary energy extraction, thereby contributing to weight gain [8].

### 3.3 Influence of gut microbes on nutrient absorption and bioavailability

Additionally, the influence of gut microbes extends to nutrient absorption, as these microorganisms can modulate the bioavailability of essential nutrients, including vitamins B and K and minerals such as iron. This modulation has far-reaching implications for host nutrition and overall health [49]. Moreover, gut bacteria display a remarkable ability to metabolize dietary polyphenols, transforming these compounds into bioactive substances with potential health benefits [50]. These advantages include anti-inflammatory and antioxidant properties, emphasizing the complex interplay between the host metabolism and gut microbiota in the goal of well-being [51].

## 4 Key metabolites produced by the gut microbiota and their potential effects on the host, including modulation of immune response and inflammation

### 4.1 SCFAs and immune modulation

SCFAs are good for health and possess anti-inflammatory properties. Their immuno-modulatory function includes promoting the differentiation of regulatory T cells and stopping the production of pro-inflammatory cytokines [52]. These metabolites are histone deacetylases (HDACs) inhibitors that promote tolerogenic and anti-inflammatory cell phenotype. It is necessary for maintaining immune homeostasis. These SCFAs activate nuclear factor - kappa B (Nk-kB) and inhibit the pro-inflammatory cytokine tumor necrosis factor (TNF) in peripheral blood mononuclear cells [53,54]. SCFAs are a crucial regulator of NF-kB activity and pro-inflammatory innate immune response. The HDAC inhibition by SCFAs also has anti-inflammatory effects on intestinal macrophages [55].

### 4.2 Vitamins synthesized by gut microbiota

Fat-soluble vitamins are vitamin A (retinol and beta-carotene), vitamin D (ergocalciferol and cholecalciferol), vitamin E (tocopherol) and vitamin K (phylloquinone, menaquinone) are transported to various tissues. These former vitamins act as signaling and bioactive effectors, while water-soluble vitamin B complex and vitamin C act as coenzymes. Vitamins alter gut bacteria, increasing biodiversity and elevating SCFA levels [56]. Furthermore, the gut microbiota synthesizes essential B vitamins, specifically folate (vitamin B9) and biotin (vitamin B7). These vitamins play indispensable roles in various metabolic processes within the host [43].

### 4.3 Amino acid derivatives and other metabolites influencing immunity

Amino acid derivatives, such as tryptophan metabolites and indole compounds, are products of gut bacteria's metabolic activity. These compounds hold sway over mood regulation and immune function [57]. Notably, indole-3-propionic acid (IPA), derived from tryptophan metabolism, exerts anti-inflammatory properties and may regulate immune responses by modulating immune reducing inflammation and cell activation [58]. Taurine and tryptophan conjugates, as microbial metabolites, emerge as influential signaling molecules with the potential to regulate immune cell activity and mitigate inflammation, particularly within the gut environment [59].

Trimethylamine-N-oxide (TMAO), another vital metabolite, is closely connected to cardiovascular health [60]. Elevated concentrations of TMAO synthesized by gut microbiota after digesting choline and carnitine in food have also been linked with chronic inflammation and arteriosclerosis. Thus, the gut microbiota plays a significant role in the system's health [61]. Polyphenol metabolites, generated through the breakdown of dietary polyphenols by gut bacteria, harbor antioxidant and anti-inflammatory properties, contributing to overall host health [51].

Urolithins, microbial metabolites of ellagic acid (found in fruits like pomegranates and berries), demonstrate anti-inflammatory and antioxidant potential, further highlighting the impact on dietary components and gut microbiota's health effects [62]. Lastly, bile acid metabolites, specifically secondary bile acids, interact with host receptors and possess the capacity to modulate immune responses and inflammation, with potential consequences for immune cell function and gut barrier integrity [63].

## 5 Microbial metabolism associated with GI cancers

### 5.1 Inflammatory metabolites and GI cancer risk

The complex link between gut microbiota and GI malignancies is complicated and has important implications for our knowledge of cancer formation and progression [64]. Persistent inflammation in the GI tract has been well-established as a significant risk factor for GI malignancies. The gut microbiota, via its metabolic functions, is pivotal in regulating inflammation [27].

### 5.2 Metabolic influences on gut epithelium and GI cancer

Certain bacterial species within the gut microbiota can produce pro-inflammatory metabolites like LPS and ROS, elevating the risk of GI cancer and fuelling inflammation [65,66]. Intriguingly, some gut bacteria can produce genotoxins, directly damaging DNA within the gut epithelium [67]. This type of DNA damage may result in genetic mutations and, in certain instances, kickstart the process of cancer formation [68]. An illustrative case involves specific strains of *Escherichia coli* that generate colibactin, a genotoxic substance with strong associations with colorectal cancer (CRC) [69].

### 5.3 Immune dysregulation, dysbiosis and GI cancers

**Fig. 3:**
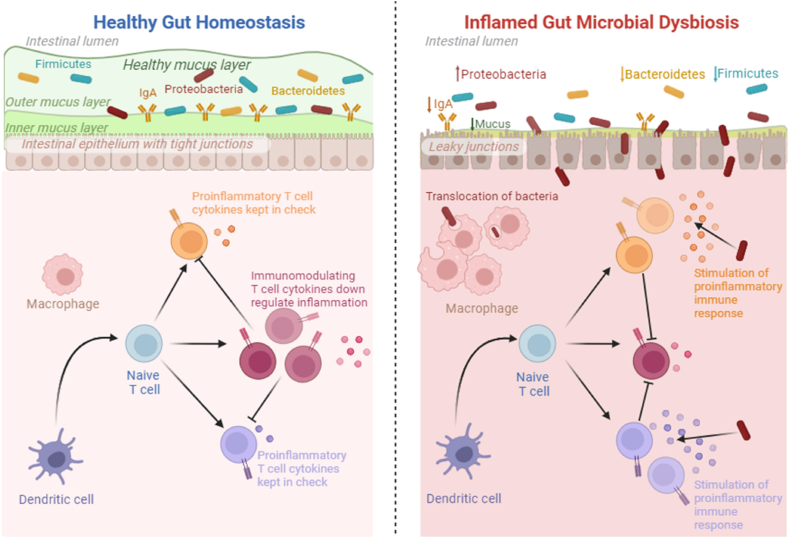
Healthy gut: balanced microbiota for well-being. Inflammation results from microbial imbalance, harming gut function.

The gut microbiota does not stop at metabolic functions; it also influences the host's immune response (Fig. 3) [17]. The alteration of gut microbial communities leads to immune dysregulation, ultimately causing autoimmune disorders [70]. Disruption of the immune response, resulting from alterations in the composition or metabolic functions of the microbiota, can substantially influence the progression of GI cancers [64]. This intimate interaction underscores the microbiota's multifaceted role in shaping the GI cancer landscape [71]. Lastly, dysbiosis can disturb the delicate balance of microbial metabolism within the gut, potentially promoting carcinogenesis [72]. The accumulating evidence has demonstrated the presence of severe dysbiosis in the intestinal microbiota of patients with GI cancer. Dysbiosis causes inflammation in the gut due to leaky junctions in the intestinal epithelium. The inflammation occurs due to the translocation of bacteria inside the epithelium, which stimulates the pro-inflammatory immune response [73].

## 6 Metabolites in gut microbiome-Mediated cancer progression

### 6.1 SCFAs and their role in cancer

Gut bacteria produce SCFAs, especially butyrate, which plays a vital role in the growth of cancer cells. They achieve this by cell death processes like apoptosis and autophagy, controlling metabolism, impacting cell behavior changes (Epithelial-mesenchymal transition process), and regulating the cell cycle. They also influence essential genes that promote or suppress tumors in various cancer types [74]. SCFAs have been shown to impact cancer progression by affecting gene expression through histone deacetylase inhibition, potentially influencing oncogenic signaling pathways [75].

### 6.2 Other microbial metabolites and their impact on cancer

Another group of metabolites, the indoleamine 2,3-dioxygenase (IDO) metabolites, produced by certain gut bacteria, activate the IDO pathway, which plays a role in immune regulation and can influence the tumor microenvironment (TME) [76]. These metabolites, such as kynurenine, can suppress the immune response against tumors [77]. Furthermore, certain gut bacteria can convert primary bile acids into secondary bile acids, such as lithocholic acid (LCA) and deoxycholic acid (DCA), which have been linked to the advancement of CRC and may alter oncogenic signaling pathways [78]. Furthermore, gut microorganisms can alter polyamine metabolism, where polyamines like putrescine and spermidine are essential for cell proliferation and dysregulation, which has been related to various malignancies [79].

### 6.3 Metabolites involved in inflammation and immune modulation

Taurine-conjugated bile acids have been linked to CRC progression and inflammation, potentially influencing the TME by modulating immune responses and inflammation [80]. Furthermore, LPS, when present in the gram-negative bacteria in the cell wall, can cause persistent inflammation, leading to cancer growth and progression. LPS promotes the production of pro-inflammatory cytokines and activates Toll-like receptors (TLRs) [81]. Another metabolite, TMAO, produced by gut bacteria from dietary precursors like carnitine and choline, has been associated with certain cancers and an increased risk of cardiovascular disease [61]. TMAO may influence cancer progression through mechanisms involving inflammation and oxidative stress [82].

### 6.4 Metabolites influencing the tumor microenvironment (TME)

Metabolites produced by microbiota also influence tumor growth by changing the environment around them. This environment, known as the TME, includes many immune cells and signaling molecules called cytokines. Microbiota-derived metabolites play a crucial role in this environment by controlling how immune cells develop and communicate [83]. Some gut bacteria create indole metabolites, which can activate the aryl hydrocarbon receptor (AhR), which is involved in immunological responses and inflammation [84]. The indole metabolites that activate AhR can potentially affect the TME [85]. These metabolites aid the complicated interaction between cancer growth and gut microbiota [86].

### 6.5 Gut microbiota composition, metabolites, and cancer types

#### 6.5.1 CRC: impact of microbiota and metabolites

**Fig. 4:**
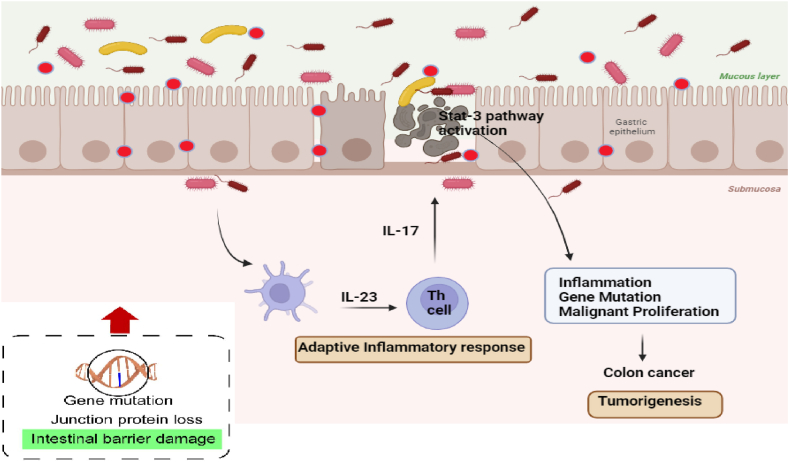
Colon cancer progression involves gut microbes speeding it up by compromising the mucosal barrier. It allows bacterial products to trigger immune responses, leading to inflammation that fuels cancer development.

For CRC, many studies have pointed to the influential role of gut microbiota compositions in promoting or inhibiting its development (Fig. 4) [87]. Notably, specific bacterial species, such as Enterotoxigenic *Bacteroides fragilis* (ETBF) and *Fusobacterium nucleatum*, have been linked to increased inflammation and the progression of CRC. These microbes are known to generate metabolites like LPS, which significantly contribute to the progression of CRC. In stark contrast, other commensal bacteria, such as fecal bacteria and various *Bifidobacterium* species, have emerged as potential protectors against CRC [64]. These beneficial microbes can create anticancer SCFAs and anti-inflammatory, such as butyrate, which are pivotal in immune response modulation and gut barrier integrity [86,88].

#### 6.5.2 Gastric cancer and the role of anti-inflammatory gut bacteria

When it comes to gastric cancer, *Helicobacter pylori*, a well-known stomach colonizer, has been identified as a critical risk factor. *H. pylori* chronic infection frequently results in chronic inflammation and the generation of ROS, culminating in DNA damage and the development of carcinogenesis [89,90]. Conversely, intriguing findings have surfaced, indicating that specific gut bacteria boasting anti-inflammatory properties, such as *Lactobacillus* and *Bifidobacterium* strains, may wield a protective influence against gastric cancer [91]. These probiotic strains exhibit the capacity to finely tune immune responses and attenuate inflammation within the gastric environment [92,93].

#### 6.5.3 Hepatocellular carcinoma and the complex microbiome-metabolite interplay

When examining liver cancer, particularly hepatocellular carcinoma (HCC), a compelling dichotomy unfolds [94]. In the gut microbiota ecology, dysbiosis has been demonstrated to stimulate the production of endotoxins and pro-inflammatory cytokines, adding to the stimulation of inflammation. Both chronic inflammation and fibrosis are pivotal risk factors in the genesis of HCC [64]. Paradoxically, specific gut bacteria hold the potential to produce metabolites like bile acids, which intricately participate in liver metabolism and can sway the risk landscape of HCC [95]. Additionally, applying probiotics and prebiotics has sparked interest in bolstering liver health and mitigating the risk of HCC in individuals grappling with liver disease [96,97].

## 7 Precision diagnosis in healthcare

### 7.1 Introduction to precision diagnosis

Precision diagnosis, also known as precision medicine or personalized medicine, is a medical diagnosis and treatment approach that takes an individual's unique genetic, environmental, and lifestyle factors into account to tailor healthcare decisions and treatments to the specific characteristics of each patient [98]. It aims to optimize medical care by considering the variability between individuals in terms of disease risk, progression, and response to treatment [99].

### 7.2 Advantages and applications of precision diagnosis in cancer

**Customized Treatment Plans:** Precision diagnosis enables healthcare practitioners to develop personalized treatment recommendations based on a patient's genetic makeup and molecular profile [100]. This method is especially essential in cancer treatment because tailored treatments can more successfully address specific genetic abnormalities [99].

**Reduced Adverse Effects:** Tailoring treatments based on a patient's genetic and physiological characteristics can help reduce adverse effects [101]. Precision diagnosis can improve patient safety by avoiding treatments that are unlikely to be effective or are known to cause harm to a particular patient [102].

**Early Disease Detection:** Precision diagnosis can enable the early detection of diseases and conditions, allowing for interventions before diseases become severe. For example, genetic screening can identify individuals at higher risk of certain diseases, enabling proactive measures or early treatment [103].

**Improved Drug Selection:** Precision diagnosis can aid in identifying the most effective drugs for specific individuals. Pharmacogenomics, for example, investigates how a person's genetic composition influences their pharmacological response, assisting in selecting the best treatment and dose for each patient [104].

**Enhanced Patient Engagement:** Precision diagnosis can empower patients by involving them in healthcare decisions. When patients understand their conditions' genetic and molecular basis and treatment options, they may become more engaged and proactive in managing their health [105].

**Cost-Effective Healthcare:** While the initial costs of precision diagnosis can be high, it has the potential to lead to cost savings in the long run by avoiding ineffective treatments, hospitalizations, and complications. It can benefit both patients and healthcare systems [106].

## 8 Microbial metabolites as biomarkers in GI cancers

### 8.1 CRC and microbial metabolites

SCFAs have been implicated in both the progression and prevention of CRC [107]. Secondary bile acids, which include LCA and DCA, are created by the gut microbiota through bile acid metabolism. Certain secondary bile acids have been linked to an increased risk of CRC and gallbladder cancer [108]. TMAO, derived from the microbial metabolism of dietary carnitine and choline, has been linked to an elevated risk of CRC [109]. Microbial metabolites such as bile acids, hydrogen sulfide, and N-nitroso compounds act as essential signaling molecules. They facilitate communication between gut microbes and the host, influencing CRC development. These metabolites could potentially serve as biomarkers for detecting and predicting the progression of CRC at an early stage [110].

### 8.2 Specific gut bacteria and GI cancer biomarkers

*F. nucleatum*, a specific gut bacterium, has been found in high abundance in CRC. It holds potential as a diagnostic biomarker by detecting its DNA and metabolites in fecal samples [111]. The fact is that higher I3A, which is a tryptophan metabolite and gastric cancer marker, appears in the urine of patients with gastric cancer, indicating its use as a marker for diagnosing this disease [112]. The metabolic products secreted by the bacteria in the stomach known as H. pylori can also act as markers for gastric cancer risk [113]. These microbial metabolites represent a promising avenue for early cancer detection and risk assessment in various GI cancers. However, ongoing research and clinical validation are essential to confirm their reliability and clinical significance [114].

## 9 Research and potential of microbial signatures in cancer diagnosis

### 9.1 Microbial signatures in CRC detection

In a study titled "*The human gut microbiome as a screening tool for CRC*," specific microbial signatures associated with CRC were identified. The findings from this study suggested the potential utility of these microbial signatures for early detection and screening of CRC [115]. Microbial species like *Anaerobutyricum halli*, *Clostridioides difficile,* and *F. nucleatum* are specific markers for excess body weight-CRC. These findings help improve the precision of diagnosing and treating CRC [116].

### 9.2 Gastric cancer and microbial biomarkers

In gastric cancer research, "*Gastric microbial community profiling reveals a dysbiotic cancer-associated microbiota*" discovered changes in the stomach microbiome of those with gastric cancer. Furthermore, the study suggested that these microbial fingerprints might be helpful in the diagnosis of stomach cancer [93]. Bacteria are significantly higher in gastric cancer patients. It indicates that bacterial overgrowth in the stomach is a possible marker for gastric cancer [117]. Gastric cancer patients have a greater variety of gastric microbiota [118]. At the phylum levels, firmicutes are significantly higher in gastric cancer patients [119].

### 9.3 Pancreatic cancer and microbial changes

A study titled "*The Pancreatic Cancer Microbiome Promotes Oncogenesis by Induction of Innate and Adaptive Immune Suppression*" identified specific microbial changes associated with pancreatic cancer. The study indicated that these microbial alterations might influence the tumor microenvironment, potentially impacting the development and progression of pancreatic cancer [120]. The microorganisms in the gut often cause the development of various types of cancer. The disruptions in the balance of these microbes have been linked to the progression and survival rates of pancreatic cancer. It expands the field of TME research in pancreatic cancer [121].

### 9.4 Microbiome and cancer in other tissues

A study titled "*Microbiota of human breast tissue*" uncovered microbiota within breast tissue, although it didn't directly establish a connection between microbial patterns and breast cancer. This finding has prompted inquiries into the potential significance of breast tissue microbiota in the context of breast cancer development, and it calls for additional exploration in this field [122].

The paper "*Interaction between the microbiome and TP53 in human lung cancer*" investigated the connection between the microbiome and TP53 mutations in lung cancer. Given the high prevalence of TP53 mutations in lung cancer, this study underscores the importance of the lung microbiome in this disease [123].

The research "*Possible role of the uterine microbiome in the onset of endometrial cancer*" primarily concentrated on endometrial cancer. Nevertheless, it also delved into the potential participation of the uterine microbiome in gynecological cancers, encompassing ovarian cancer, thereby paving the way for additional investigations in this field [124].

## 10 Microbiota-targeted interventions in GI cancers

### 10.1 Introduction to microbiota-targeted interventions

Microbiota-targeted interventions offer promising avenues for influencing therapeutic approaches to GI cancers [125]. FMT is a procedure that includes introducing fecal material from a healthy donor into a patient's GI tract in order to reestablish a balanced microbiota. A study unveiled the potential of FMT to enhance cancer immunotherapy [126]. Findings revealed that patients with metastatic melanoma responding to immunotherapy possessed a higher abundance of specific gut bacteria, and FMT from these responders into non-responders improved treatment outcomes [127].

### 10.2 Probiotics, prebiotics, and dietary interventions

Probiotics, live microorganisms conferring health benefits when consumed, and prebiotics, compounds promoting beneficial gut bacteria growth, have demonstrated remarkable potential in cancer therapy [128]. A randomized controlled trial illustrated that advanced lung cancer patients receiving a combination of immunotherapy and a specific probiotic strain experienced improved response rates and longer progression-free survival compared to those undergoing immunotherapy alone [129]. Additionally, dietary fiber, categorized as prebiotics, was found to modulate the gut microbiome and potentially reduce CRC risk [130].

Dietary therapies show great potential for influencing the gut microbiome and, as a result, the risk of GI cancer [131]. According to research, dietary fiber consumption improved the efficiency of immunotherapy in melanoma patients by increasing the development of good gut bacteria [132]. Furthermore, postbiotics, metabolic byproducts of probiotics, and microbiota-derived metabolites have garnered attention for their potential to exert direct or indirect effects on cancer cells [133]. Summarizing their findings, the authors explored the ability of metabolites originating from the microbiota, such as SCFAs, to impact immune reactions and hinder the progression of CRC [134].

### 10.3 Antibiotics and microbiota dysbiosis

Antibiotics, known for their capability to alter gut microbiota composition, have emerged as a crucial factor influencing cancer risk and treatment responses [135]. Research findings revealed a correlation between the usage of antibiotics and decreased effectiveness of immune checkpoint inhibitors in individuals with cancer. It underscores the possible implications of antibiotic-induced dysbiosis on the immune milieu within the gut [136]. Lastly, microbiota-based diagnostics have emerged as an essential tool for detecting GI malignancies early [137]. Research underscored the potential of the gut microbiota's composition as a non-invasive biomarker for early CRC detection, highlighting microbiota studies' groundbreaking impact on cancer diagnosis [138].

## 11 Microbial metabolites and their role in drug metabolism

### 11.1 Microbial metabolites and cytochrome P450 enzymes (CYPs)

Microbial metabolites can exert a profound stimulus on drug metabolism, culminating in many consequences for drug efficacy and safety [139]. One significant facet of this influence revolves around their capacity to modify drug metabolism, mainly impacting the liver's cytochrome P450 enzyme (CYP) activity [140]. For instance, an illustrative study published in the prestigious journal "Science" in 2013 illuminated how microbial metabolites like TMAO can effectively inhibit the activity of CYP2D6, an indispensable enzyme responsible for metabolizing various drugs [61].

### 11.2 Activation and inactivation of drugs by gut bacteria

The activation or inactivation of drugs by gut bacteria represents a pivotal mechanism by which microbial metabolites can alter drug behavior [141]. A prodrug is an inactive drug that converts into an active form after metabolism. The liver performs most of the metabolic processes and produces the enzymes by the gut microbiota, which activates the conversion of the prodrug to its active form. This active form of the drug can lose its therapeutic efficacy due to the deactivating action of the gut microbial enzymes [142]. Notably, these microorganisms can transform prodrugs into their active forms or deactivate drugs, as exemplified by converting the anticancer drug irinotecan into its active metabolite SN-38 through microbial enzymatic activity [143].

### 11.3 Modulation of drug bioavailability

Microbial metabolites can alter medication bioavailability by modulating gut permeability and influencing drug absorption [144]. This effect is exhibited by microbial metabolites such as SCFAs, which can improve medicine absorption by altering the gut environment [145]. The gut microbiota can modulate the bioavailability of oral drugs by being involved in their biotransformation, influencing the drug transport process, and altering some GI properties. They modulate the metabolism of bile acids like microbial bile salt hydrolase (BSH) enzymes and microbial 7 alpha-dehydroxylases [146].

### 11.4 Influence on immune responses and inflammation

It is also essential to consider how microbial metabolites can impact our immune system's response and inflammation regulation. It, in turn, can have a substantial effect on the effectiveness and safety of a variety of medications [147]. A notable example of this phenomenon is the use of immune checkpoint inhibitors for cancer therapy, where the impact of the gut microbiota can either bolster or impede treatment results [148]**.**

### 11.5 Production of toxic compounds and drug interactions

Gut microbiota can produce a range of metabolic responses to drugs and xenobiotics. This results in both direct effects on drug metabolism and toxicity. The indirect effects on host metabolic enzymes/transporters/immune system along with disposition and competition of bacterial-derived metabolites for xenobiotic metabolism pathways. It gives both the intended and unwanted effects of the drug. These responses raise the risk of producing toxic compounds [149]. Microbial metabolites can give rise to the production of toxic compounds that may interact with drugs, exacerbating their toxicity [150]. An illustrative example is the invention of p-cresol by certain gut bacteria, which has the potential to interact with drugs like acetaminophen, thus potentially increasing their hepatoxicity [151].

### 11.6 Complexities in drug-drug interactions

It is essential to recognize that microbial metabolites can introduce complexities in drug-drug interactions by altering the pharmacokinetics of multiple drugs [152]. These interactions have the potential to result in unforeseen side effects or modifications in drug efficacy, underscoring the intricate interplay between microbiota, microbial metabolites, and drug therapy [153]. For example, drug-drug interaction between venlafaxine and propafenone in which venlafaxine is primarily metabolized by CYP2D6 and substrate of P-gp (permeability glycoprotein), while propafenone is a known substrate and inhibitor of both CYP2D6 and P-gp. Therefore, propafenone may induce an increase in venlafaxine plasma concentration with the development of hallucinations [154].

## 12 Advancements and future Prospects in microbiota-based therapies

### 12.1 Microbiota-based therapies in GI diseases

FMT successfully treats recurrent *C. difficile* infections, highlighting the importance of gut microbiota in disease presentation and treatment results [155]. Another approach is to utilize prebiotics and probiotics to modify the gut microbiota by encouraging the development of beneficial bacteria [156].

Furthermore, personalized microbiome modulation involves tailoring microbiota-based therapies to an individual's unique gut microbiome composition and specific disease profile, opening up new possibilities for enhancing treatment outcomes [157].

### 12.2 Gut microbiota and immunotherapy

The gut microbiota influences the effectiveness of immunotherapy mainly by regulating the immune response. It can influence innate and adaptive immunity through its metabolites [158]. SCFAs are essential metabolites produced by gut microbiota, and they have the potential to modulate the immune system [159]. The gut microbiota biomarkers can change with different forms of immunotherapy. For example, metagenomic shotgun sequencing and unbiased metabolomics profiling identify specific gut microbiota and metabolites linked to checkpoint therapy in melanoma conditions [160]. The clear connection between the gut microbiota and cancer immunotherapy's effectiveness and potential side effects underscores the need to explore how microbiota composition might influence responses to immune checkpoint inhibitors [148].

### 12.3 Diet and gut microbiota

Diet is one of the most potent modulators of gut microbiota for their functions and composition. This interaction also affects the immune system and intestinal barrier, emphasizing the crucial role of diet in developing and treating various diseases [161]. The effect of nutrition on the gut microbiota has been investigated as a method of potentially improving treatment effects. The research revealed that even brief dietary changes can substantially transform the gut microbiota composition, highlighting the adaptability of this intricate microbial community concerning health and disease [162]. Dietary fiber or probiotics help improve the gut microbiome, which can enhance human health and may help reduce obesity [163].

## 13 Future Directions and challenges

Studying gut microbiota and its role in GI cancers is a complicated study; it requires more in-depth investigation. However, this is a very complex area due to some significant impediments. Firstly, it is pretty challenging to point out the microbial population in each individual, as microbiota diversity makes an inconsistent association with colon cancer. In addition, it is difficult to separate the causal connection from simply correlation because nobody knows if changes in microbiota precede or follow cancer progression. However, more complications emerge because of diet and lifestyle issues. These require separating the environmental effects from issues like food consumption and exercise. However, it is pretty tricky to find a specific bacterial species of those hundreds in the gut microbiome and identify their exact mechanism of causing gastric cancer.

Technical issues such as the accuracy and expense involved in sampling and analytical techniques pose a threat of bias and compromise the interchangeability of research outcomes. Researchers must be cautious while dealing with ethical data and personal health records, for its scrutiny may lead to ethical challenges or even infringement on individual's rights, including violation of privacy. While longitudinal studies provide the total picture of microbiota-cancer interactions across time, they also take enormous effort, often involving keeping tabs on participants for an extended period. The research involves the animal model, which doubts its relevance to humans' physiological functioning, considering their distinct biological properties. However, applying research findings to real-world applications like diagnostics and treatment requires stringent verification, which may take years for people to gain. Also, this aspect involves the multifactorial nature of GI cancers arising out of genetic backgrounds and environmental interaction with microbes.

Advanced methodologies like metagenomics are necessary for identifying specific microbial functions and metabolic pathways involved in cancer development, and considerations regarding ethnic and geographic variability add another layer of complexity due to differences in gut microbiota composition across diverse groups and regions. In light of these challenges, standardized research methods and the conduct of large-scale clinical trials are indispensable in ensuring the accuracy, reproducibility, generalizability, and ethical considerations of research outcomes and facilitating regulatory approval within medical and scientific research.

## 14 Conclusion

This comprehensive data review highlights the intricate relationship between gut microbiota and GI cancers, shedding light on various facets of this complex connection. GI cancers, which present a substantial global health burden, are influenced by lifestyle choices, infections, and, as emphasized in this review, the gut microbiota. The gut microbiota, a diverse community of microorganisms inhabiting the digestive tract, plays a crucial role in human health. It contributes to digestion, nutrient absorption, and the regulation of the immune system. Dysbiosis, or an imbalance in the gut microbiota, can lead to various health issues, including autoimmune diseases, metabolic disorders, and potential impacts on mental health.

Importantly, this review underscores the therapeutic potential of harnessing the gut microbiota to promote health and treat various conditions. Interventions such as prebiotics, probiotics, and fecal microbiota transplantation are under exploration in this context. The review primarily focuses on the metabolic interactions between the gut microbiota and GI malignancies. The data suggests that the gut microbiota's metabolic activities can significantly influence the onset, progression, and response to treatment of GI cancers. This insight holds promise for advancing precision diagnosis and innovative therapeutic approaches in GI cancer treatment.

Understanding and leveraging the power of the gut microbiota may pave the way for more effective and personalized disease-fighting treatments. Furthermore, the study offers a broader perspective on the importance of gut microbiota in overall health, emphasizing its impact on numerous aspects of human physiology, including digestion, immunology, and even mental well-being. This data review underscores the intricate and multifaceted nature of the gut microbiota's impact on human health, specifically focusing on its significant role in GI cancers and the potential for groundbreaking advancements in diagnosis and treatment through microbiota-based approaches.

## Funding

This work was supported by the 10.13039/501100001809National Natural Science Foundation of China (32070671, 32270690).

## Consent for publication

All the authors approved the final version of the manuscript.

## Ethics approval and consent to participate

"Not applicable".

## Data availability statement

Data included in the article/supp. Material/referenced in the article. This article is based on the literature available on public platforms like PubMed, Scopus, and Google. Relevant data will be shared once the request will be received through proper channels. As no original research data has been generated, so, we have not deposited it in any publicly available repository.

## CRediT authorship contribution statement

**Wen Xie:** Writing – original draft, Visualization, Investigation, Formal analysis, Data curation. **Aditi Sharma:** Writing – original draft, Visualization, Investigation, Formal analysis, Data curation. **Hitesh Kaushik:** Writing – review & editing, Formal analysis, Data curation. **Lalit Sharma:** Writing – review & editing, Formal analysis. **Nistha:** Writing – review & editing, Formal analysis. **Md Khalid Anwer:** Writing – review & editing, Formal analysis. **Monika Sachdeva:** Writing – review & editing, Formal analysis. **Gehan M. Elossaily:** Writing – review & editing, Formal analysis. **Yingbo Zhang:** Writing – review & editing, Visualization, Formal analysis. **Ramkumar Pillappan:** Writing – review & editing, Formal analysis. **Maninderjit Kaur:** Writing – review & editing, Formal analysis. **Tapan Behl:** Writing – review & editing, Validation, Supervision, Project administration, Funding acquisition, Formal analysis, Conceptualization. **Bairong Shen:** Writing – review & editing, Supervision, Funding acquisition, Formal analysis, Conceptualization. **Rajeev K. Singla:** Writing – review & editing, Supervision, Formal analysis, Conceptualization.

## Declaration of competing interest

The authors declare that they have no known competing financial interests or personal relationships that could have appeared to influence the work reported in this paper.
